# Traditional Birth Attendance (TBA) in a health system: what are the roles, benefits and challenges: A case study of incorporated TBA in Timor-Leste

**DOI:** 10.1186/s12930-014-0012-1

**Published:** 2014-11-26

**Authors:** Decio Ribeiro Sarmento

**Affiliations:** School of Public Health, Georgia State University, Atlanta, 30303 Georgia

**Keywords:** Traditional birth attendants, Community health workers, Reproductive health, Family health promoters in Timor-Leste

## Abstract

**Background:**

One current strategy to overcome the issue of shortage of qualified health workers has focused on the use of community health workers in the developing countries to deliver health care services specifically to the most vulnerable communities in the rural areas. Timor-Leste is the one of the world’s newest developing countries that has incorporated the traditional birth attendance in its health system through a family health promoter initiative in response to reproductive and child health, hence to improve primary health care delivery and increase number of healthcare workforce.

**Methods:**

The study utilized a non-systematic review of the literature using key words such as community health workers, traditional birth attendants, reproductive health, child health and health outcomes. A case study from Timor-Leste was also used.

**Results:**

Traditional birth attendants have performed wide variety of tasks including outreach and case finding, health and patient education, referrals, home visits and care management. Evidence indicated that there were, to varying degrees, positive associations between traditional birth attendance training and maternity care. Traditional birth attendance training was found to be associated with significant increases in attributes such as knowledge, attitude, behavior, advice for antenatal care, and pregnancy outcomes. However, some challenges faced by traditional birth attendants’ role in encouraging women to go to health center for preventive services would be the compliance and refusal of the referral. The implementation case study from Timor-Leste shows that integrating traditional birth attendance into a national healthcare system through Family Health Promoter program has been programmatic effective. It is recommended that the implementation should consider regular communication between health staff and community leaders in recruiting members of family health promoters, and the use of supportive supervision tools to identify weaknesses in the management of this initiative.

**Conclusion:**

In Timor-Leste, incorporating traditional birth attendance through family health promoter program has played crucial roles in delivering and increasing access to reproductive health services by women in rural communities of the nation. Whilst it requires a long-term commitment and good partnership, the current reduction in maternal mortality ratio in Timor-Leste is encouraging and serves to illustrate how this initiative aims to improve primary health care delivery and increase number of healthcare workforce.

## Background

Human resources are the key ingredients to a successful health system functioning [1]. Nonetheless, a large number of countries are still facing an overall shortage of qualified health workers to perform and deliver the primary health care services [2]. According to the World Health Organization (WHO), most developing countries still experience varying degrees of shortages in qualified health workers in which current estimates suggest there is a shortage of 4.2 million health workers worldwide [3]. A greater number of strategies have then been established to overcome this issue. Those strategies include improving salaries and working conditions for qualified health professionals, providing financial and compensation incentives, and increasing capacity building for formal health workers [4]. The WHO report also states that latter strategy to respond to the widespread problem of human resources for health has focused on the use of community health workers (CHWs) in the developing countries where formal health workers are too few to deliver health care services specifically to the poorest and most vulnerable communities in the rural and remote areas.

The initial concept of CHWs has been used for several decades to render certain basic health care services to underserved populations [5]. CHWs have been defined as the lay members of community who work exclusively to serve people who have lacked access to adequate care, and establish vital links between health care consumers and providers to promote health in community settings [6,7]. It seems likely that the concept of CHWs and activities they perform mainly depend upon the local context and situation. In specific response to the reproductive and child health, these community-based workers sometimes referred to as the traditional birth attendants (TBAs) in some situations, have played important role in providing maternity care based on the local community needs. It is imperative to further the focus on the TBAs and the activities that these cadres of health workers deliver in relation to reproductive and child health. Therefore, this paper summarizes the evidence-based information for the inclusion of TBAs in a health care system in response to reproductive and child health services and provides a case example from Timor-Leste of how it has been implemented.

## Methods

The case study utilized a non-systematic review of the literature using key words such as community health workers, traditional birth attendants, reproductive health, child health and health outcome. A structured search of PubMed, MeSH, PMC and Medline was also performed for studies published from 1990 up to March 2014. The search terms were limited to English-language publication, and due to limitation of published literatures on community health workers in Timor-Leste, the search terms were not limited to other sub-regional groups obtained from different developing countries. To try and capture reports in the grey literature, Google and key development agency websites were also searched. Articles were included if they provided some level of systematic review of evidence such as meta-analysis and observational studies relating to community health workers in rural settings of low and middle-income countries.

A specific case study from Timor-Leste was utilized for the discussion of incorporating TBAs in a health care system. This case study does not include any experimental research nor any research carried out on humans and animals and as such did not require an ethical approval.

## Results

### Basic roles of CHWs

CHWs have been defined as the members of community who are selected by the communities to provide care for a broad range of health issues including reproductive and child health to the poorest and most vulnerable communities [8]. Before describing the overall performance of CHWs, it is necessary to identify the different terms used for CHWs in different countries across the globe (Table 1).

**Table 1:** **Alternative title for CHWs in various countries**

**Title**	**Country**
*Activista*	Mozambique
*Agente comunitario de salud*	Peru
*Agente comunitário de saúde*	Brazil
*Anganwadi*	India
*Animatrice*	Haiti
*Barangay* health worker	Philippines
Basic health worker	India
*Brigadista*	Nicaragua
*Colaborador voluntario*	Guatemala
Community drug distributor	Uganda
Community health agent	Ethiopia
Community health promoter	various countries
Community health representative	various countries
Community health volunteer	Malawi
Community health worker	various countries
Community nutrition worker	India
Community resource person	Uganda
Female community health volunteer	Nepal
Female multipurpose health worker	Nepal
Health promoter	various countries
*Kader*	Indonesia
Lady health worker	Pakistan
Maternal and child health worker	Nepal
*Monitora*	Honduras
Mother coordinator	Ethiopia
Outreach educator	various countries
Paramedical worker	India
*Promotora*	Honduras
Rural health motivator	Swaziland
*Shastho shebika*	Bangladesh
*Shastho karmis* (leaders of *shastho shebika*)	Bangladesh
*Sevika*	Nepal
Traditional birth attendant	various countries
Village drug-kit manager	Mali
Village health helper	Kenya
Village health worker	various countries

Adapted from Lehmann and Sanders [8].

The establishment of CHWs has apparently been associated with the growing shortages of human resources within a health system, and the functions of CHWs are mainly to help addressing this widespread issue. Despite of being known by different titles, some literatures reveal that the tasks performed by CHWs are essentially the same. A review study conducted in the year of 1998 demonstrates that CHWs have performed wide variety of tasks including outreach and case finding, health and patient education, referrals, home visits and care management [9]. CHWs help connect health care services with local communities for the provision of counseling, health education, treatment and advocates experienced in local needs [10]. For example, in the rural settings of Gambia, Ghana, South Africa and Tanzania, CHWs with minimal additional training have played vital roles in delivering counseling, treatment and health education for such important diseases as malaria, HIV and tuberculosis for the local communities [10]. Indeed, in the South Asia countries like Bangladesh, Bhutan, Nepal and Sri-Lanka, CHWs can help health care systems overcome both personnel and financial shortages by providing high quality and cost-effective services to people in their homes [11]. Therefore, the most probable view on this would be that by delivering basic health care services to communities, CHWs have improved adherence to treatment and reduced the burden of time and financial on both health care consumers and providers. Nevertheless, the tasks performed by CHWs may vary enormously by settings and community needs. In many rural settings of the developing countries, CWHs sometimes referred to as TBAs, may have also been selected by communities to represent a major source where pregnant women cannot access to maternal health services due to cultural, socioeconomic or geographic barriers [12].

### Benefits of CHWs in response to reproductive and child health services

In many poor parts of the world where human resources are still in very short supply to provide basic health care, CHWs have remained a significant workforce in delivering reproductive and child health services. In specific to TBAs, they have been widely defined as the community or family members (normally females of old age groups) who are a product of tradition in assisting mothers during home delivery [13]. The competence and skills of TBAs may vary widely across settings. Following the years since the introduction of the Safe Motherhood Program, training of TBAs has been recognized as one of the interventions intended to reduce maternal mortality and morbidity rates as well as to improve the reproductive health of women [14]. A greater number of public health studies have been undertaken to review and identify the explanations for continued existence of TBAs, hence their roles in delivering maternity care within the poorest communities.

One purpose from previous literatures is mainly focus on the identification of the effectiveness of TBA training and its impact on reproductive health outcomes. A series of meta-analysis review identified that there were, to varying degrees, positive associations between TBA training and maternity care [14]. TBA training was found to be associated with significant increases in attributes such as TBA knowledge, attitude, behavior and advice for antenatal care [14] and pregnancy outcomes [15]. Also, TBA training was significantly associated with referral behavior and maternal service use by women with obstetric complications [16]. These associations may lead to a significant reduction in maternal mortality and morbidity. However, although TBA training may have been associated with reduction of a substantial proportion of maternal mortality and morbidity, the information and material about the training program should be improved and the effect of the training needs to be evaluated in order to develop a strong evidence base.

Using the same parameters such as knowledge, attitudes and practices, another systematic review also demonstrated that TBAs had incorporated the information from the training program into their knowledge and practice for prevention, recognition and management of postpartum hemorrhage (PPH) in Gambia [17]. It was revealed that despite of reducing PPH morbidity and mortality in home births in this setting, there is a need for Gambian TBAs to be trained to implement other practices relevant to prevention of this pregnancy complication in the primary care setting. Similarly, assessments in Brazil, Guatemala and Indonesia have shown that TBAs can identify early signs of complications during labor and delivery, and successfully refer mothers for treatment in health centers by skilled health workers [18]. From this evidence, it seems likely that training of TBAs may result in large improvements in reproductive and child health, hence contribute to substantial reduction of maternal morbidity and mortality.

In addition to performing home delivery and referral system for pregnancy complications, TBAs have also been engaged in some basic health care functions such as health promotion and disease prevention. In Sub-Saharan Africa where home births remain a strong preference and might be often the only option, TBAs have been involved in health promotion and disease prevention [19]. It is indicated that TBAs have played some critical roles in health promotion by preventing mother to child Human Immunodeficiency Virus (HIV) transmission in the region where HIV prevalence is remarkably high [19]. Those tasks performed by TBAs have included disseminating information about perinatal HIV transmission, identifying pregnant women in their communities and facilitating the use of available antenatal care and maternity care, ensuring routine HIV counseling for women and their partners, supervising treatment of mother and infant with nevirapine and offering advice to women on reducing the risk of HIV transmission [19].

In contrast to those findings above, it is argued that trained TBAs without the support of skilled back-up services may not significantly reduce maternal mortality ratio [17]. A recent cluster-randomized controlled trial study in Pakistan demonstrated that involving trained TBAs in reproductive care did not reduce maternal mortality, but instead it led to a reduction in perinatal mortality [19]. The findings showed that a cluster-adjusted odds ratio for perinatal death was 0.70 for intervention sub-districts as compared to control, which suggested a significant reduction in perinatal mortality of about 30% in the intervention group [20]. Therefore, it is likely clear to assume that integrating TBA training into the health care system could be an effective strategy to reduce perinatal mortality, but not maternal mortality ratio.

Similarly, training of TBAs in hygienic practice during delivery does not prevent postpartum infections [21]. The findings from a study that took place in a rural area of Bangladesh illustrated that although trained TBAs were likely to practice hygienic delivery, there was no significant difference in levels of postpartum infections when delivering births by trained TBAs and untrained TBAs [21]. It seems likely that the inclusion of clean practice in TBA training program is ineffective to prevent postpartum infections. The implication related to postpartum infections might be due to possible determinants such as nutritional factors of the women particularly micronutrients and vitamins deficiencies [21]. Therefore, postpartum infection would not be affected directly by clean practices at delivery performed by trained TBAs [21]. The most probable view on this would be that TBA training in hygienic practice does not seem to give impact to the improvement of women reproductive health; therefore, evaluation of the training program is rigorously required with special attention to measure the specific outcomes such as maternal morbidity due to postpartum infections.

### Practical challenges

Involving trained TBAs in reproductive health care has several advantages, including ease of access to reproductive care and delivery of care to women in the community. However, challenges should be taken into consideration in order to measure limitations in delivering reproductive care. Some barriers of TBAs’ role in encouraging women to go to health center for preventive services would be the compliance and refusal of the referral. The reasons might lie within the financial limitation, lack of transportation and patients’ fear of painful treatment from formal health workers [22]. Indeed, it seems likely that appropriate training and supervision should be provided for TBAs [15]. The appropriate training simply means that it has to be practical-based training. When appropriate training, such as clean practice of cutting umbilical cord (clean labor practice), is available, these cadres of community health workers can serve as effective as readily available human resources in mobilizing communities, and delivering reproductive care to women in the poorest settings. This would ultimately be applicable to poorest settings where TBA training has not yet been considered as part of an innovative strategy to combat shortages of human resources in health systems. It is recommended that supervision and direct observation of TBAs at work are critical for clarification of the most appropriate role of TBAs in reducing maternal mortality and morbidity in the poorest and most vulnerable settings. Therefore, the roles of TBAs should be functioned by good referral system and sufficient health infrastructures, equipped with clean-birth kits after training sessions and supported from professionally trained health workers during regular supervision [21].

### Incorporating TBAs in practice: a case study from Timor-Leste

#### Demographic

Timor-Leste is located in Southeast Asia and Pacific, northwest of Australia and the east end of the Indonesian archipelago. The country has a land area of 14,954 square kilometers and a population of over one million. The country’s boundaries include the eastern half of the island of Timor, the Oecusse enclave in West Timor and the islands of Atauro and Jaco. Timor-Leste gained its independence after 25 years of Indonesian occupation through a referendum made in September 1999. More than half of the Timorese are under 18 years of age. In purely economic terms, Timor-Leste is a middle-income economy and one of the most oil dependent economies in the world [23]. The high fertility rate, where on average women give birth to 5.7 children throughout their lifetime is a key contributing factor to the high annual population growth rate of 2.7 per cent [24]. While much of the country remains agrarian, a phenomenon of rapid urbanization has been reported where about 22 per cent of the population lives in the urban areas [25]. The health status at the community level remains low and for many children and women life remains a day-to-day struggle for survival.

#### Maternal and child health situation in Timor-Leste

Maternal height and pre-pregnancy weight has enormous influence over birth outcomes. Shorter and lighter women are more likely to have babies with low birth weight. These women are also most likely to experience difficulties in childbirth and could likely die. Maternal and under-five mortalities remain high: maternal mortality is 557 per 100,000 live births and under-five mortality is 64 per 1,000 live births [24]. Statistics also show that 30 per cent of births are delivered by a skilled nurse or midwife, nearly 18 per cent of deliveries are carried out by TBAs and 49 per cent by a relative or some other people, which are relatively high [24]. Whilst much have been done to build the capacity of the health sector service delivery, it is clear that the quality of services needs to be further enhanced.

#### Healthcare resources in Timor-Leste

Timor-Leste significantly faces some challenges in generating resources for health systems particularly human and physical resources such as facilities and equipment. Current workforces in Timor-Leste still suffer shortages for the provision of health services nationwide [26]. Table 2 below illustrates the distribution of national health workers in Timor-Leste in 2011.

**Table 2:** **Distribution of national health workers in Timor-Leste**

**Category**	**Number**
*Assistant Pharmacists*	116
*Environmental Health Officers*	27
*Laboratory assistants*	132
*Medical Practitioners (General Practitioner)*	75
*Medical Practitioners (Medical Specialists)*	9
*Medical technicians*	48
*Midwives*	53
*Nurses*	1800
*Nutrition Professionals*	31
*Optometrists*	13
*Pharmacists*	15
*Physiotherapists*	1
*Public Health Officers*	13
*Public Health Professionals*	64
*Sanitary inspectors*	160
*Traditional Birth Attendants (Community Health Volunteers)*	1647

**Figure 1:**
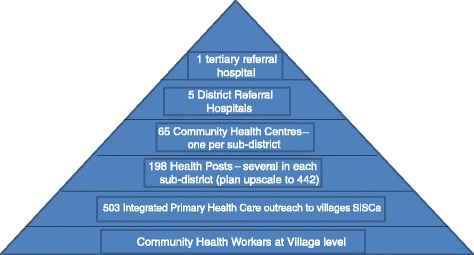
**Overall structure of the Timor-Leste Health System.** Adapted from Martins and Trevena [27].

In terms of health structure, the organization for national health system consists of central services, and district health services (community health centers, health posts and mobile clinics) (Figure 1) [27]. Relatively, physical resources (facilities) in the district level are still inadequate and human resources have limited capacity to provide health care services to all people. Assistance from external organizations and individuals are needed and welcomed. The Ministry of Health of Timor-Leste has collaborated with Cuban government by employing around 350 Cuban medical doctors to supplement clinical services in district and sub-district levels and sending around 680 East Timorese students to study Medicine under the scholarship from Cuban government [28].

### Incorporating TBAs through family health promoters initiative in Timor-Leste

In 2007 the Ministry of Health (MoH) of Timor-Leste rolled out a national cadre of volunteer Family Health Promoters or Promotor Saude Familia (PSF) with main objective to increase the number of health workers and strengthen its national health system in delivering maternal and child health services including reproductive health intervention program into the community level. This initiative has been extensively supported by development agencies, particularly the government of the United States through its Agency for International Development (USAID).

PSFs are community health volunteers that are not MoH staff nor do not receive a monthly salary for their work, however, they are provided with incentives. Community members are selected to be PSFs by village leadership and trained by MoH staff to provide basic community health services with an emphasis on teaching and motivation of families they visited, including reproductive health. Especially in rural areas, some of the volunteers include TBAs who have played a key role in assisting pregnant women before, during and after delivery. These volunteers are also expected to take an active role in conducting health promotion activities through an integrated community health services (also called SISCa). SISCa program has been developed to address the shortfall of health care service delivery at the community level by improving uptake of preventive healthcare and access to basic medical services [27,29]. One responsibility of PSFs is to conduct home visits and assist health staff when they provide community outreach services. In the year of 2009, the number of trained PSFs registered in the database of MoH was over 1,500 [30]. The PSF program has been extended along with the introduction of SISCa.

PSFs have been provided with training, materials and supervision in their house-to-house visits with pregnant mothers in a campaign called Mai Ita Koko (or Come Let’s Try) [30]. The type of training included basic knowledge to encourage women to attend clinics for pre- and post-natal care and practice of handling labour with clean equipment. The training was provided by group of skilled health care workers from district level in conjunction with support from some Non-Governmental Organizations (NGOs) such as Care International and World Vision.

**Figure 2:**
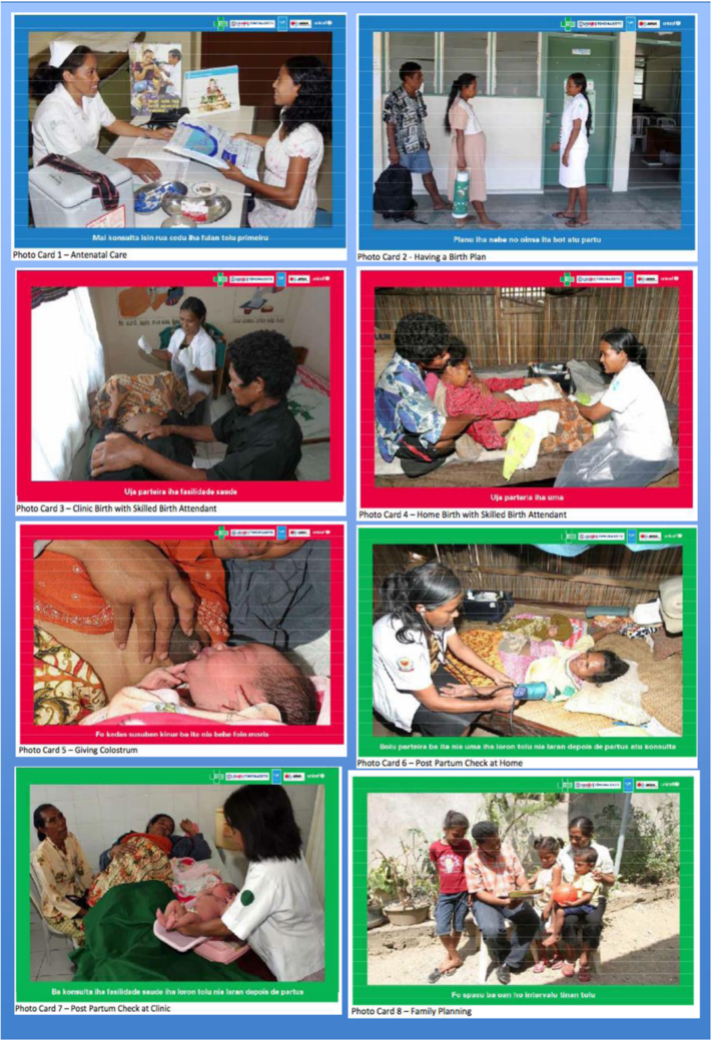
**Photo cards developed for PSFs to use during implementation.** Adapted from Health Alliance International (HAI) [30].

In the training, eight photo cards that depict relevant Timorese images and portray recommended maternal behaviours were developed (Figure 2). The images and messages on the cards are simple, clear, culturally relevant, and action oriented. PSFs have been trained to use the cards as an educational tool during home visits and to encourage women and families to adopt one or more behaviours, such as having a skilled birth attendant or selecting a family planning method after delivery. The chosen behaviour(s) is checked off on a colourful poster that contains each of the photos and is left with the family to remind them of their choice.

The PSFs follow up with families throughout the mother’s pregnancy to monitor progress toward their goals. The home visits have been well received by mothers who appreciate the support provided by the PSFs. Since the implementation of PSF program, a significant improvement has been observed in the proportion of women receiving antenatal care from a skilled health staff as a result of PSF referral system – an increase from 41 per cent in 2006 to 86 per cent in 2010 [24]. The maternal mortality ratio has also improved from 660 per 100,000 live births in 2003 to 557 per 100,000 live births in 2010.

It seems likely that the PSF is both programmatic and cost effective. There are some strength related to the recruitment, training, implementation and management of this program. They include MoH is able to recruit many PSFs, MoH is able to train PSFs, PSFs actively participate in SISCa program, and SISCa provides solid structure to manage PSFs. Although being effectively programmatic, there are some specific challenges faced by PSFs during implementation in Timor-Leste. A major challenge has been the compliance and refusal of the referral due to financial limitation and lack of transportation for patients who reside in rural areas. Another challenge has been the minimum incentives provided to PSFs, which contributes to lack of motivation to provide outreach activity program.

Even though many PSFs actively participate in monthly implementation of SISCa, continuation to socialize, supervise and promote the role of PSFs is required to address the constraints from this initiative as mentioned above. Therefore, it is recommended that effective implementation should consider regular communication between health staff and community leaders in recruiting PSFs, and the use of supportive supervision tools to identify weaknesses in the management of this initiative. A proper evaluation program should also be conducted to further examine the effectiveness of the PSF initiative.

## Conclusion

Despite of being the most important aspect of health care systems, human resources have been a neglected component of a health system development. It is imperative that the engagement of CHWs in the health system function should be considered to address the growing shortage of formal health workers. This paper has identified that although the evidence of whether the roles of TBAs may reduce maternal mortality ratio varies by findings, training of TBAs is considered to be an effective complimentary strategy to attend home births that still remain high in the poorest countries and in the poorest rural populations within countries. Indeed, this paper has described how Timor-Leste has integrated TBA through PSF program into its basic health service package delivery to reduce reproductive and child health issues of the country.

It is evident that TBA training has shown significant results in reduction of maternal mortality and morbidity in some developing countries where home delivery is high and still the main preference. Findings from some literatures illustrate that substantial improvement in health outcomes has been achieved and sustained through the TBA training. It shows that trained TBAs have provided basic level of maternity care, for example, their role in prevention and management of postpartum haemorrhage. Also, the greatest contribution from trained TBAs to reduce maternal mortality and morbidity can be observed in the area of health promotion, for example, their role in disseminating information to women on perinatal transmission of HIV. In delivering reproductive care to women in the community, TBAs may face various challenges such as referral compliances that should be considered. In specific to Timor-Leste, incorporating TBAs through PSF initiative has played crucial roles in delivering and increasing access to reproductive health services by women in rural communities of the nation. Those volunteers have been trained to recognize complications of labour and make appropriate referrals. Whilst it requires a long-term commitment and good partnership, the current reduction in maternal mortality ratio in Timor-Leste is encouraging and serves to illustrate how this initiative aims to improve primary health care delivery and increase number of healthcare workforce. This concept may be linked to Timor-Leste being on track for many of the health Millennium Development Goals (MDGs) with the need for a more concerted effort in immunization coverage, HIV/AIDS education and malaria control. In addition, strengthening the current system will hopefully help to achieve these targets that will be a major achievement for one of the world’s youngest independent nations.

Therefore, it is well worthwhile to explore the possibilities of engaging TBAs in primary health care and of training them accordingly. Last but not least, this whole concept of this paper highlights that to achieve the MDGs number four and five; there is a need to ensure the effectiveness and appropriateness of reproductive and child health services delivered to communities by trained TBAs. In so doing, access to reproductive care must be supported by sufficient health system functions, particularly human resources, referral system and basic infrastructures.

## Abbreviations

CHW: Community health worker
HIV: Human Immunodeficiency Virus
MDG: Millennium Development Goal
MoH: Ministry of Health
NGOs: Non-Governmental Organizations
PPH: Post-Partum Hemorrhage
PSF: Promotor Saude Familia (or Family Health Promoter)
SISCa: Servisu Integradu da Saude Communitaria (Integrated Community Health Services)
TBA: Traditional birth attendance
TBAs: Traditional birth attendants
UNICEF: United Nations Children’s Fund
USAID: U.S. Agency for International Development
WHO: World Health Organization
